# Notes and News

**Published:** 1894-12-22

**Authors:** 


### Notes and News.

COLLECTED AND COMMUNICATED.

The Empress Frederick has forwarded to Mr. Holt, the secretary of the Throat Hospital, Golden Square, a bust of the late Prince Waldemar of Prussia, to be placed in the ward which is to be named after him.

The anniversary of the Shrewsbury Infirmary has just been celebrated. The institution has attained an age of nearly 150 years, and is the centre of much interest after a long and useful existence. We are happy to learn that its financial condition is satisfactory.

The Aberdeen Royal Infirmary is progressing towards the possession of a new convalescent home, which will greatly add to the completeness of its work. The trustees of the estate of the late Mr. John Chalmers, of Banchory, have given £5,000 for the new home and £2,000 towards the building fund, and £3,000 towards the endowment.

A new and commendable direction for generosity is suggested to "wrong doers" by following the example of one of themselves. The "wrong doer" we refer to has subscribed 6s. to the Salisbury Infirmary in acknowledgment of his claims to the title. It would certainly be an act of grace, and a benefit to our charities, if all misdoings were acknowledged in a like manner, the anonymous nature of the offering making it devoid of unpleasantness to the giver.

We feel constrained to offer a few words of praise and appeal on behalf of the work amongst orphans accomplished by Miss Sharman. Miss Sharman has no committee. Whilst strikingly free from the usual defects of an individual enterprise, Miss Sharman's work is full of personal enthusiasm and activity, which show astonishing results. The headquarters are at the Orphans' Home in West Square, Southwark. Then there are country branches at Tunbridge Wells and Gravesend. Miss Sharman does not possess a house for the children at the sea, but it is her great wish to do so. The children are supported at the moderate average cost of £15 per annum, a return any manager of an orphanage might feel satisfied with. Miss Sharman collected £4,759 during the past year. All her accounts are carefully put out and examined, and trustees assist her in her finances.

The Local Government have consented to allow the Epsom Joint Hospital Board to erect a hospital for infectious diseases at a site on the chalk near Banstead Downs. The consent was given contingent on proper means for the filtration of sewage being adopted.

Through the generosity of the family of the late Mr. George Burt, Swanage is to possess a new cottage hospital, larger and more complete than the existing building, which also owed its existence to the liberality of the same family. The hospital is to be erected to the memory of the late Mr. G. Burt and his wife, by their children. New rules are to be framed; and doubtless this occasion will be taken to study the rules of the best institutions of the kind, foremost amongst which rank the arrangements at the Harrow Cottage Hospital.

It has been suggested that the use of blood as a curative agent was on the increase in Paris. This, however, we learn on reliable authority, is not the case, although a certain number of persons still resort to the abattoires every morning to partake of this nauseous form of so-called cure. Blood-baths are also used, and patent medicines are sold under the name of "poudre de sang" and other titles. French doctors, we are told, are not often responsible for prescribing any of these forms of treatment.

Proof of the unsectarian character of the management of St. Catherine's Home for patients with incurable diseases, at Bradford, is given by the recent report of the inmates treated there during the past year. Of these 20 were members of the Church of England, 4 Roman Catholics, 4 Congregationalists, 2 Baptists, 1 Methodist, and 2 who did not claim to belong to any religious denomination. The cases included in this period 22 sufferers from cancer, 7 from consumption, 1 non-malignant tumour, 2 paralysis. Applications for admission to this well-managed home far exceed the number of beds, and therefore until considerable structural additions are found feasible, vacancies are always filled by the most suffering and the poorest of the candidates.

It is interesting to note the progress of the Hospital Sunday and Saturday Movement throughout the English speaking world. We have just been sent the results of the first Hospital Saturday Fund Collection in Sydney. The whole amount collected was £1,949, and of this £1,426 was collected in the streets. The small result from the weekly collections, as the secretary points out, is due to the movement only having been instituted a few months, besides which workers are more scattered than they are in large towns in England. The cost of the collection was £121, or about 7 per cent. The Fund has been registered as a limited company, and the articles of association provide that every friendly or trade association contributing £1, shall be entitled to elect one delegate, and every committee contributing shall be entitled to elect their honorary secretary as a delegate, and further the Board of the fund shall have the power to elect amongst the delegates any person whom they may deem suitable. The fund has power to assist the medical charities of the whole of New South Wales.

